# Ophthalmic Manifestations Revealing Neurological Relapse in T-cell Acute Lymphoblastic Leukemia: A Case Report

**DOI:** 10.7759/cureus.79687

**Published:** 2025-02-26

**Authors:** Loubna Mouhib, Hala Ait Ammar, Mounia Bendari, Mehdi Khamaily, Mohamed Elbelhadji

**Affiliations:** 1 Department of Ophthalmology, Mohammed VI International University Hospital, Mohammed VI University of Sciences and Health (UM6SS), Casablanca, MAR; 2 Department of Hematology, Mohammed VI International University Hospital, Mohammed VI University of Sciences and Health (UM6SS), Casablanca, MAR

**Keywords:** chorioretinal infiltration, neurological relapse, optic nerve infiltration, papilledema, t-cell acute lymphoblastic leukemia

## Abstract

The ophthalmic manifestations of acute leukemia are critically important, as ocular involvement may be the sole clinical indicator of the disease or signify a recurrence of leukemia. Ocular manifestations include the orbit, anterior segment, optic nerve, retina, and choroid, with posterior segment involvement being the most prevalent.

We report the case of a 19-year-old woman with acute lymphoblastic leukemia (ALL). The patient was in remission after completing the Group for Research on Adult Acute Lymphoblastic Leukemia (GRAALL) protocol. A decrease in visual acuity revealed an infiltration of the anterior and posterior segments of the patient’s eye, indicating a disease relapse. A cerebral magnetic resonance imaging (MRI) showed substantial optic nerve infiltration. A lumbar puncture confirmed the recurrence, with a positive result for leukemia. It was a relapse of ALL, unmasked by the ophthalmic involvement, which prompted a change in disease status and treatment.

Ocular involvement can sometimes be the first sign of the disease or its recurrence, making it crucial to recognize and address promptly, as it may precede other serious clinical manifestations. Therefore, it is essential to systematically include an ophthalmological examination in the follow-up of patients with acute leukemia.

## Introduction

Acute leukemia (AL) is a type of hematological cancer in which infiltrates of clonal, proliferative/poorly differentiated hematopoietic cells occupy the bone marrow, blood, and other tissues [1].

Ophthalmic manifestations of AL are pivotal, as ocular involvement might be the only clinical sign revealing the disease or its recurrence. These manifestations arise from oculo-orbital invasion by immature hematopoietic cells (blasts), direct extension from the central nervous system (CNS), or vascular and rheological complications. They may also result from side effects of both local and systemic treatments [2].

We report the case of a 19-year-old woman with acute lymphoblastic leukemia (ALL) in remission after completing the Group for Research on Adult Acute Lymphoblastic Leukemia (GRAALL) protocol, in whom a decrease in visual acuity revealed infiltration of the anterior and posterior segments of the eye, indicating a relapse of the disease.

## Case presentation

We present the case of a 19-year-old woman with two years history of T-lymphoblastic acute leukemia (T-ALL). The initial form of the disease was characterized by leukocytic tumor burden, which responded well to corticosteroid therapy and achieved remission after induction of the GRAALL protocol. She reached the third maintenance cycle, according to GRAALL protocol, during which the patient reported acute unilateral vision loss.

**Figure 1:**
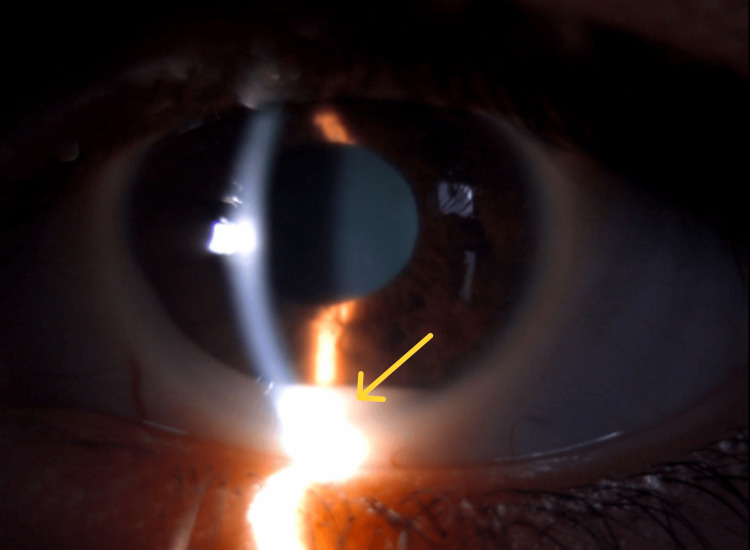
Slit-lamp photograph showing tumor pseudohypopyon (yellow arrow)

Ophthalmic examination showed an asymmetric visual acuity of 20/20 in the right eye and counting fingers in the left eye. Intraocular pressure (IOP) was within normal limits bilaterally (oculus dexter (OD): 15 mmHg; oculus sinister (OS): 16 mmHg). The anterior segment examination demonstrated a tumor pseudohypopyon (Figure 1) with significant anterior chamber cellular reaction (2+ Tyndall). Additionally, focal iris elevation suggested underlying tumor infiltration.

**Figure 2:**
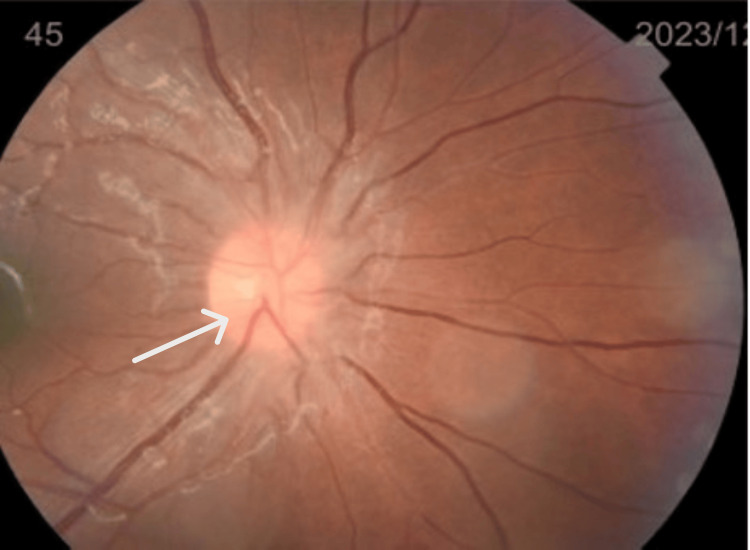
Stage 1 papilledema (white arrow) in the right eye

**Figure 3:**
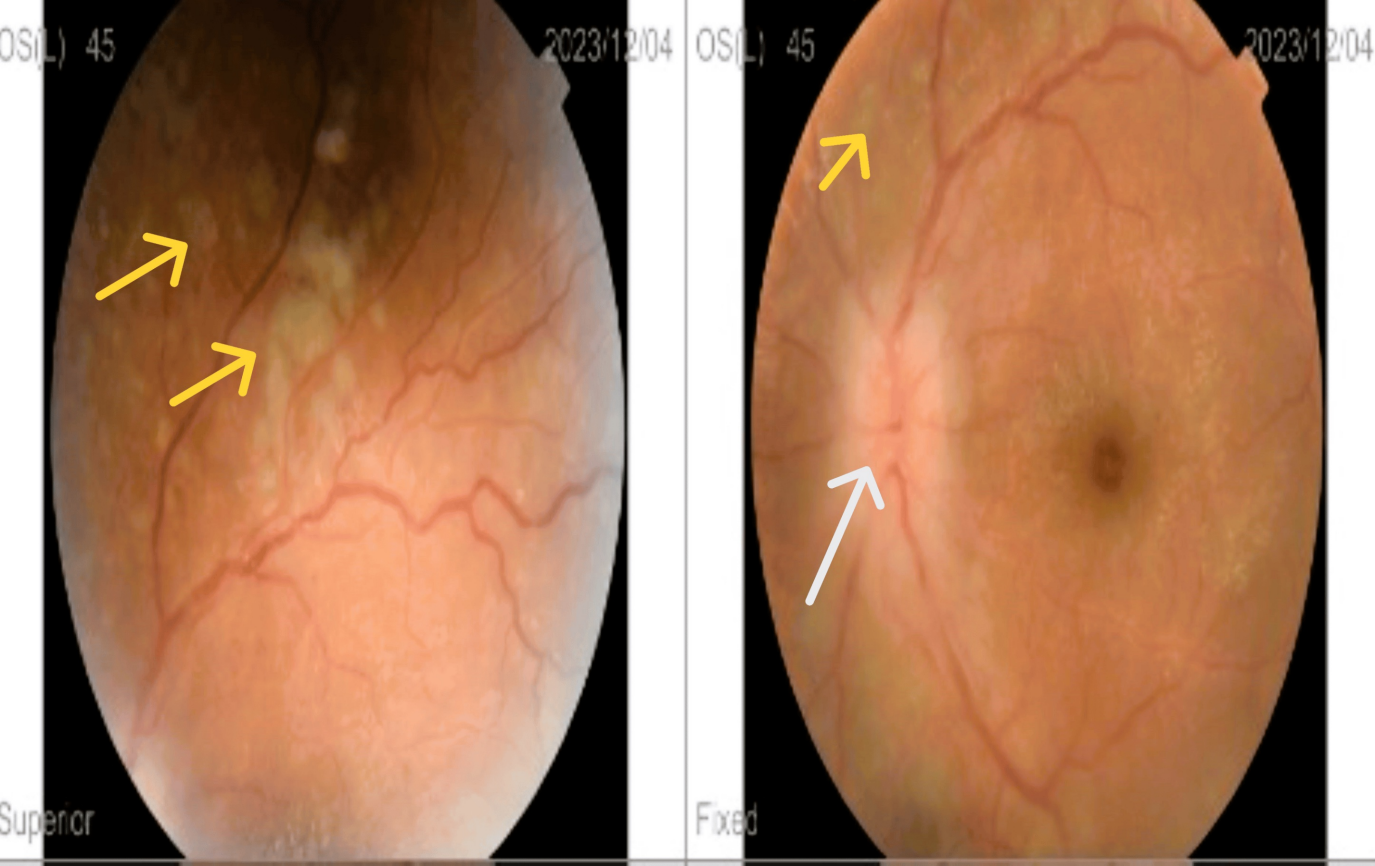
Stage 2 papilledema (white arrow) in the left eye with chorioretinal infiltration (yellow arrows)

**Figure 4:**
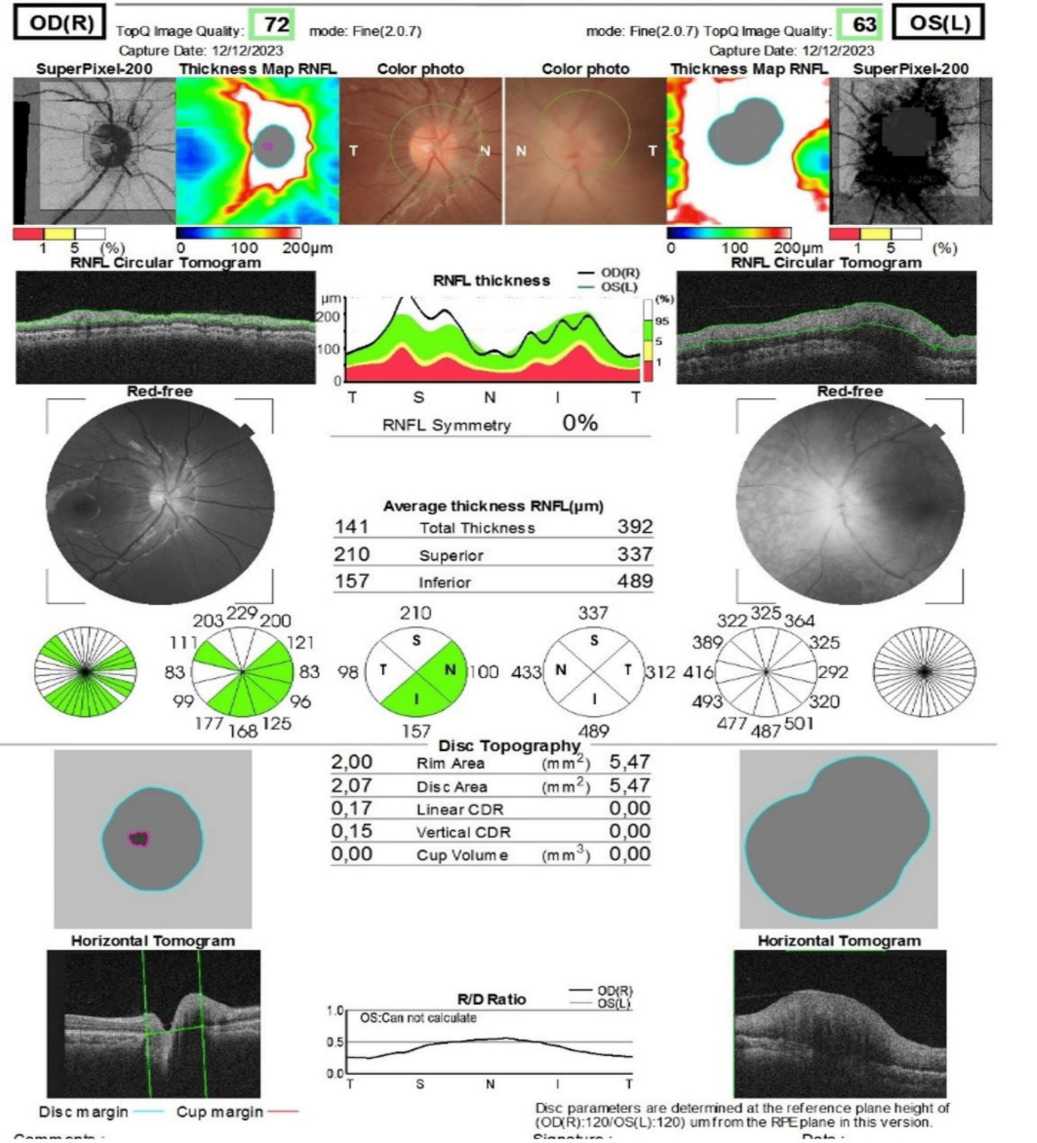
Papillary spectral domain optical coherence tomography (OCT): superior and temporal papillary thickening in the right eye and total thickening in the left eye

**Figure 5:**
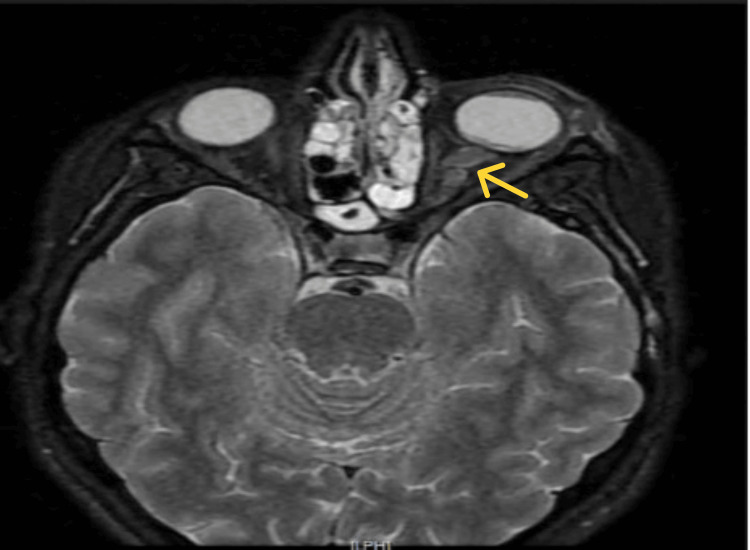
Brain and orbital MRI: tortuous and thickened appearance of the optic nerve (yellow arrow)

Fundoscopic examination revealed stage 1 papilledema in the right eye (Figure 2) and stage 2 papilledema with diffuse chorioretinal infiltration in the left eye (Figure 3). The papilledema was confirmed by papillary optical coherence tomography (OCT) (Figure 4). Orbital and cerebral MRI demonstrated extensive optic nerve infiltration (Figure 5), while subsequent lumbar puncture confirmed CNS relapse with significant leukemic involvement (500 leukocytes/mm³).

Following a multidisciplinary oncology board meeting, the patient received a combination of radiation and chemotherapy. At the end of the radiation therapy cycle, a follow-up cerebral MRI showed significant improvement in the optic nerve infiltration. The ophthalmological examination revealed a reduction in the hypopyon, although papilledema remained unchanged.

The patient's clinical course deteriorated due to multiple complications, including hyperleukocytosis with tumor lysis syndrome, acute renal failure, metrorrhagia, and respiratory distress. The patient ultimately passed away due to these complications. The primary factor identified in the clinical decompensation was an antibiotic-resistant *Klebsiella* infection.

## Discussion

Ocular involvement in AL is a relatively common phenomenon, although its prevalence varies considerably between studies [1]. This heterogeneity is associated with advancements in treatments, particularly chemotherapy and preventive strategies against CNS localizations [3]. Ocular structures can be affected extensively, including the orbit, anterior segment, posterior segment, optic nerve, retina, and choroid, with posterior segment involvement being the most prevalent [4].

Although the anterior segment is less frequently involved, it may present with distinctive clinical features, such as iris heterochromia, keratouveitis with hypopyon, or spontaneous hyphema [5]. Infiltration of this segment is more frequently observed in ALL than in acute myeloblastic leukemia (AML) [5]. It could be explained by a phenomenon known as pharmacological sanctuary, where this segment partially escapes the action of systemic chemotherapy [6]. While isolated anterior segment involvement remains rare, it should not be overlooked in the follow-up of patients with ALL [5].

Ocular involvement may also result from colonization by blast cells, either by direct extension from the CNS or via the bloodstream. This invasion can take various forms, ranging from retinal hemorrhage to choroidal infiltration, sometimes associated with exudative retinal detachment [7]. These ocular lesions may indicate a medullary or meningeal relapse, underlining the importance of regular ophthalmological surveillance for early detection of these signs [7,8].

In the management of AL, ocular involvement, particularly in the case of ALL, is an unfavorable prognostic factor due to the high risk of relapse in the bone marrow or meninges [9]. Prophylactic strategies such as intensification of chemotherapy and cytostatics intrathecal injections are used to limit these risks [9,10].

Finally, ocular involvement may be the initial sign of the disease or its recurrence, potentially preceding other serious clinical manifestations, and therefore should not be neglected [11]. It is essential to incorporate an ophthalmological examination in the follow-up of AL patients to ensure early detection and appropriate management of associated ocular and neurological complications.

## Conclusions

Ophthalmic manifestations of AL may precede systemic involvement and aid in early diagnosis. These often asymptomatic signs require regular ophthalmologic surveillance, as ocular involvement can indicate recurrence or progression of the disease, particularly in ALL. Since lumbar punctures are systematically performed throughout treatment to monitor CNS infiltration, we propose including ophthalmological examinations in ALL treatment protocols. Contrary to current practice, where ophthalmological exams are only conducted when ocular symptoms arise, we advocate for regular ophthalmological monitoring. This proactive approach would enable early detection of ophthalmic manifestations and enhance patient management.

## References

[1] Laimon DN, Sakr DH, Atef B, Shaaban Y (2024). Highlights of ophthalmological manifestations in newly diagnosed acute leukemia: a correlation with hematological parameters. Ann Hematol.

[2] Ralph Rosenthal A (1983). Ocular manifestations of leukemia: a review. Ophthalmology.

[3] Paul S, Short NJ (2022). Central nervous system involvement in adults with acute leukemia: diagnosis, prevention, and management. Curr Oncol Rep.

[4] Soumya S (2023). Study of ocular manifestations in leukemia: a cross-sectional study. Int J Acad Med Pharm.

[5] Yu AM, Chan SC, Iordanous Y, Padmore RF, O'Connor MD (2019). Anterior segment infiltration of acute lymphoblastic leukemia: case report and systematic review. Can J Ophthalmol.

[6] Mateo J, Ascaso FJ, Núñez E, Peiro C, González G, Cristóbal JA (2011). Ophthalmological manifestations in acute lymphoblastic leukemia. Novel Aspects in Acute Lymphoblastic Leukemia.

[7] Hui VWK, Szeto SKH (2025). Clinical and imaging features of leukemic retinopathy. Leukemia - From Biology to Clinic.

[8] Hafeez MU, Ali MH, Najib N, Ayub MH, Shafi K, Munir M, Butt NH (2019). Ophthalmic manifestations of acute leukemia. Cureus.

[9] Ferenchak K, Dao LN, Dalvin LA (2021). Ocular relapse of B-cell acute lymphoblastic leukemia. JAMA Ophthalmol.

[10] Pui CH, Jeha S (2007). New therapeutic strategies for the treatment of acute lymphoblastic leukaemia. Nat Rev Drug Discov.

[11] Talcott KE, Garg RJ, Garg SJ (2016). Ophthalmic manifestations of leukemia. Curr Opin Ophthalmol.

